# MicroRNA-26a suppresses epithelial-mesenchymal transition in human hepatocellular carcinoma by repressing enhancer of zeste homolog 2

**DOI:** 10.1186/s13045-015-0229-y

**Published:** 2016-01-06

**Authors:** De-Ning Ma, Zong-Tao Chai, Xiao-Dong Zhu, Ning Zhang, Di-Hua Zhan, Bo-Gen Ye, Cheng-Hao Wang, Cheng-Dong Qin, Yi-Ming Zhao, Wei-Ping Zhu, Man-Qing Cao, Dong-Mei Gao, Hui-Chuan Sun, Zhao-You Tang

**Affiliations:** Liver Cancer Institute, Zhongshan Hospital, Fudan University, Shanghai, 200032 China; Key Laboratory of Carcinogenesis and Cancer Invasion (Fudan University), Ministry of Education, Shanghai, People’s Republic of China; Department of General Surgery, Changzheng Hospital, Second Military Medical University, Shanghai, People’s Republic of China; Department of Liver Surgery, Fudan University Shanghai Cancer Center, Cancer Hospital, Shanghai, People’s Republic of China

**Keywords:** microRNA-26a (miR-26a), Hepatocellular carcinoma (HCC), Enhancer of zeste homolog 2 (EZH2), Epithelial-mesenchymal transition (EMT)

## Abstract

**Background:**

Our previous study reported that microRNA-26a (miR-26a) inhibited tumor progression by inhibiting tumor angiogenesis and intratumoral macrophage infiltration in hepatocellular carcinoma (HCC). The direct roles of miR-26a on tumor cell invasion remain poorly understood. In this study, we aim to explore the mechanism of miR-26a in modulating epithelial-mesenchymal transition (EMT) in HCC.

**Methods:**

In vitro cell morphology and cell migration were compared between the hepatoma cell lines HCCLM3 and HepG2, which were established in the previous study. Overexpression and down-regulation of miR-26a were induced in these cell lines, and Western blot and immunofluorescence assays were used to detect the expression of EMT markers. Xenograft nude mouse models were used to observe tumor growth and pulmonary metastasis. Immunohistochemical assays were conducted to study the relationships between miR-26a expression and enhancer of zeste homolog 2 (EZH2) and E-cadherin expression in human HCC samples.

**Results:**

Down-regulation of miR-26a in HCCLM3 and HepG2 cells resulted in an EMT-like cell morphology and high motility in vitro and increased in tumor growth and pulmonary metastasis in vivo. Through down-regulation of EZH2 expression and up-regulation of E-cadherin expression, miR-26a inhibited the EMT process in vitro and in vivo. Luciferase reporter assay showed that miR-26a directly interacted with EZH2 messenger RNA (mRNA). Furthermore, the expression of miR-26a was positively correlated with E-cadherin expression and inversely correlated with EZH2 expression in human HCC tissue.

**Conclusions:**

miR-26a inhibited the EMT process in HCC by down-regulating EZH2 expression.

## Background

Liver cancer (mostly hepatocellular carcinoma [HCC]) is the sixth most prevalent cancer worldwide [1], and it is the second most frequent cause of cancer-related death in men and the sixth most frequent in women [2]. Surgical resection provides a cure in only about 20–30 % of HCC patients, and the reported 5-year survival rate is 40–50 %, with a high incidence of postoperative recurrence [3, 4]. Postoperative recurrence remains a major problem in reducing patient survival [5], and it may occur because of intrahepatic metastasis from the original primary tumor even for the patients with early-stage cancer [3] or from de novo carcinogenesis [6]. The precise mechanisms involved in metastasis are still not well understood, but accumulating evidence suggests that an epithelial-mesenchymal transition (EMT)-like process plays a major role [7]. EMT is a transient and reversible switch from a polarized and epithelial phenotype to a fibroblastoid or mesenchymal cellular phenotype, with the latter showing highly motile and invasive properties [8, 9]. In general, this process is a fundamental event both in the early stage of embryonic development and in pathophysiological situations, including wound healing, chronic inflammation, and carcinoma progression [7]. Several studies have reported that functional loss or down-regulation of E-cadherin is a hallmark of EMT [10, 11], and transcriptional control of E-cadherin during EMT seems to be required. Although EMT entails the down-regulation of other epithelial-specific genes, such as those for components of tight and gap junctions or desmosomes, E-cadherin down-regulation is thought to be inherent to EMT [12]. MicroRNAs (miRNAs) are an evolutionarily conserved family of small (a class of 22-nucleotide) noncoding RNAs, known to be potent modifiers of gene expression at a posttranscriptional level. They regulate multiple cellular processes such as tumorigenesis and metastasis by down-regulating gene expression in various malignancies, including HCC [13]. microRNA-26a (miR-26a), a member of the miR-26 family, has been considered to be a potential tumor suppressor in HCC [14, 15]. miR-26a has been reported to suppress tumor growth and metastasis of HCC through the interleukin (IL)-6-Stat3 signaling pathway [16], and down-regulation of miR-26a in tumor tissue was associated with poor overall survival for patients who underwent curative surgery for HCC [17]. In our previous study, we found that miR-26a indirectly suppresses tumor progress in HCC by inhibiting tumor angiogenesis and suppressing intratumoral macrophage infiltration [14, 18]. However, how miR-26a modulates tumor progression in a direct manner remains poorly understood. It was reported that miR-26a mediated E-cadherin expression, the hallmark of EMT, in oral tongue squamous cell carcinoma, and the enhancer of zeste homolog 2 (EZH2) was the presumed key molecule that mediated miR-26a-induced down-regulation of E-cadherin expression [19]. However, whether miR-26a plays a crucial role in EMT regulation and its underlying mechanism in HCC remains unknown. In the present study, we aimed to investigate the functional role of miR-26a in EMT and consider its potential as a therapeutic target in HCC.

## Results

### Down-regulation of miR-26a is associated with EMT in tumor cells

**Fig. 1:**
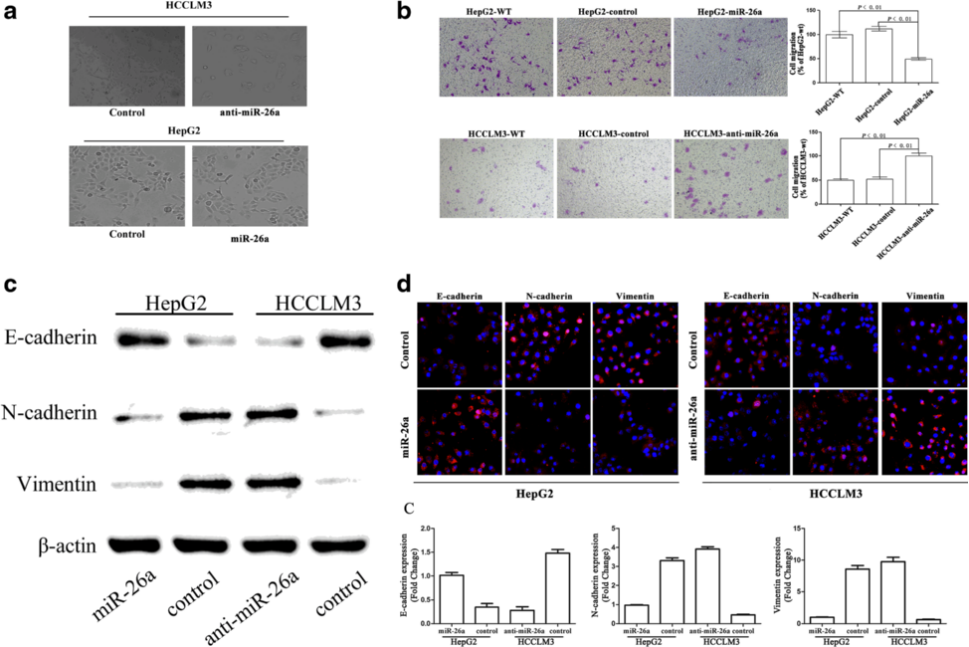
Down-regulation of miR-26a induced EMT in hepatoma cells in vitro. **a** Morphologic changes consistent with EMT (spindle-shaped cells with loss of polarity and increased intercellular separation) were observed in the HCCLM3-anti-miR-26a cells compared to control cells; HepG2 cells did not show morphological changes. **b** Cell migration through a Transwell chamber was compared between hepatoma cells transfected with miR-26a and control. The number of migrated cells was evaluated by counting 10 random fields at ×100 magnification. Western blot (**c**) and immunofluorescence assays (**d**) showed that down-regulation of miR-26a resulted in down-regulation of E-cadherin and up-regulation of N-cadherin and vimentin

In our previous study [18], the expression levels of miR-26a were modified in two human hepatoma cell lines, HepG2 and HCCLM3, and subclones with stable expression of miR-26a were established. These subclones were HepG2-wt (wild type of HepG2), HepG2-control (HepG2 transfected with the negative control of precursor miRNA), and HepG2-miR-26a (HepG2 transfected with pre-miR-26a), and HCCLM3-wt (wild type of HCCLM3), HCCLM3-control (HCCLM3 transfected with the negative control of anti-miRNA-LNAs), and HCCLM3-anti-miR-26a (HCCLM3 transfected with anti-miRNA-LNAs against miR-26a). The HCCLM3-anti-miR-26a cells gained an EMT-like phenotype compared with HCCLM3-control cells, showing an irregular fibroblast-like shape instead of a typical epithelial cobblestone appearance; HepG2-miR-26a cells had no morphological changes compared to HepG2-control cells (Fig. 1a). In the cell migration assay, HepG2-control cells exhibited significantly higher migration potential than HepG2-miR-26a cells, while HCCLM3-anti-miR-26a cells exhibited significantly higher migration potential than HCCLM3-control cells (Fig. 1b). Western blot (Fig. 1c) and immunofluorescence assays (Fig. 1d) demonstrated that miR-26a up-regulated the expression of epithelial cell marker E-cadherin and down-regulated the expression of the mesenchymal cell markers N-cadherin and vimentin. These results indicate that down-regulation of miR-26a induced EMT in HCC cells.

### miR-26a regulated EMT by targeting EZH2

**Fig. 2:**
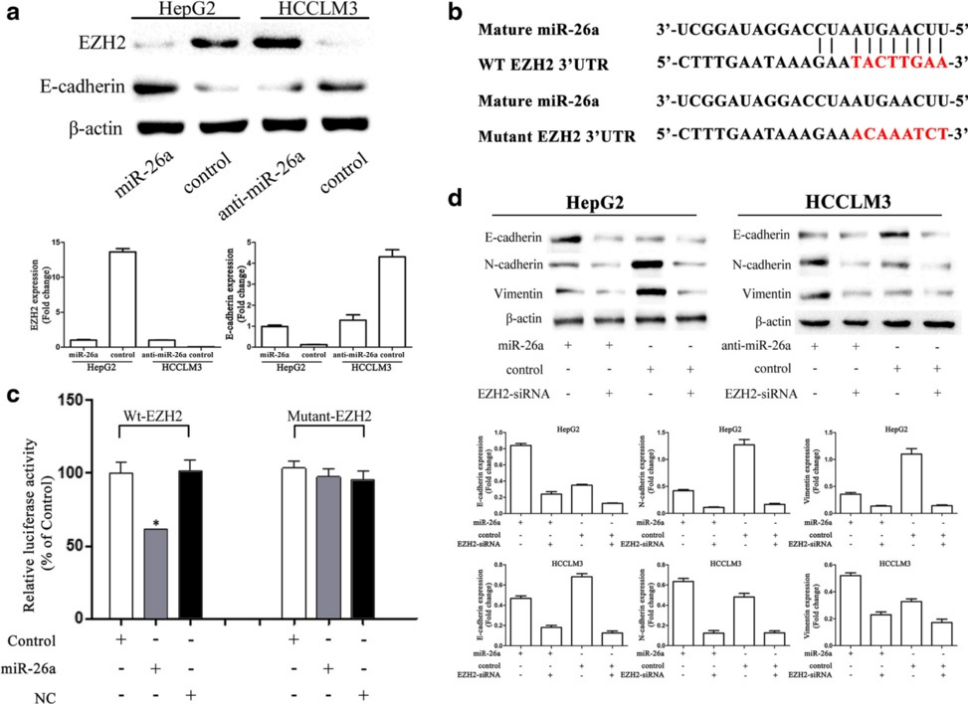
EZH2 played a critical role in miR-26a modulating EMT process. **a** Western blot assay showed that miR-26a down-regulated EZH2 expression and up-regulated E-cadherin expression. **b** Diagram of the putative binding sequence of miR-26a in the 3′-UTR containing reporter constructs of EZH2 is shown. **c** Luciferase reporter assays showed that miR-26a significantly decreased the luciferase activity of wild-type EZH2 but not mutant EZH2. NC, the mock of precursor miRNA, *P < 0.05 compared with control. **d** EZH2 siRNA transfection in HCC cells eliminated the difference in E-cadherin miR-26 overexpression and down-regulation

Overexpression of EZH2 has been found to correlate with tumor aggressiveness, metastasis, and poor prognosis in numerous cancer types [20, 21], including HCC [22]. miR-26a was reported to suppress tumor growth by targeting EZH2 in some tumors [23, 24]. To explore the mechanisms of miR-26a in moderating tumor growth and metastasis of HCC tumors, several assays were performed. Western blot analysis showed that EZH2 expression was decreased after transfection with pre-miR-26a in HepG2 cells and increased after down-regulation of miR-26a in HCCLM3 cells compared to respective controls, while the expression of E-cadherin showed the opposite results (Fig. 2a). Moreover, luciferase reporter assay was performed to determine whether miR-26a directly regulated EZH2 expression in HCC cells. The target sequence of EZH2 3′UTR (wt 3′UTR), which contained a miR-26a putative binding site, could be mutated, or a mutant sequence (mt 3′UTR) messenger RNA (mRNA) was cloned downstream of the luciferase reporter gene vector (Fig. 2b). A significant decrease of luciferase activity was observed when the cells were transfected with miR-26a compared to the control (Fig. 2c, left). The activity of the mt 3′UTR vector was unaffected by a simultaneous transfection with miR-26a (Fig. 2c, right). These results suggest that miR-26a directly interacted with EZH2 mRNA and inhibited the expression of EZH2.

Because EZH2 reportedly down-regulates E-cadherin expression [19, 25], we transfected our two HCC cell lines with EZH2 small interfering RNA (siRNA) to explore whether miR-26a could regulate E-cadherin expression by directly targeting EZH2 expression. The results show that EZH2 siRNA reduced E-cadherin expression and eliminated the difference in E-cadherin expression between HepG2-control and HepG2-miR-26a (Fig. 2d, left) and between HCCLM3-control and HCCLM3-anti-miR-26a (Fig. 2d, right).

### miR-26a inhibited tumor growth and EMT in vivo

**Fig. 3:**
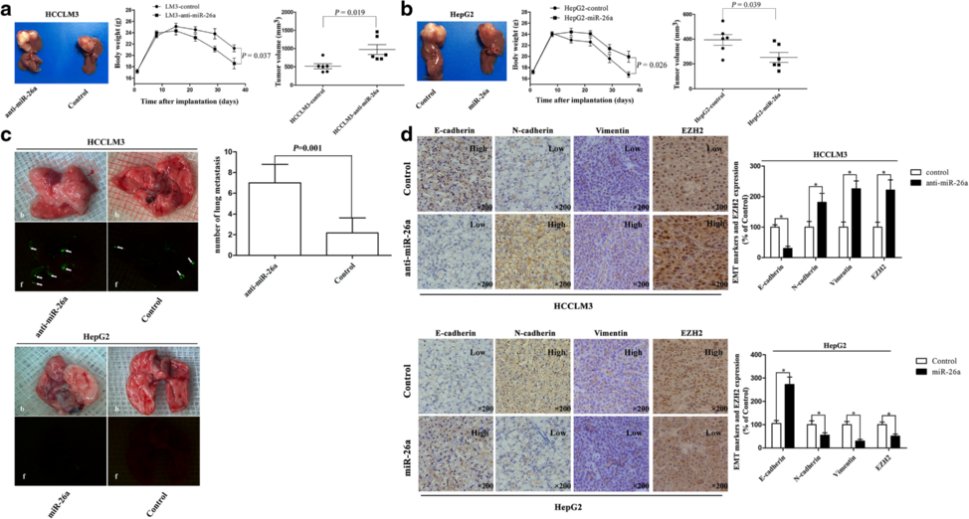
miR-26a inhibited EMT process in vivo. **a**, **b** HCC subcutaneous tumor tissues (HCCLM3-control, HCCLM3-anti-miR-26a, HepG2-control, and HepG2-miR-26a) were implanted into nude mouse livers to establish the xenograft HCC models. Thirty-five days after implantation, the mice were euthanized, and their body weights and tumor volumes were assessed. **c** Quantification of bioluminescence of lung metastatic foci showed that down-regulation of miR-26a significantly accelerated pulmonary metastasis (*arrows* indicate metastatic foci in lung). HCCLM3-anti-miR-26a and HCCLM3-control tumors were compared by both bright field (*b*) and fluorescence (*f*) imaging. No pulmonary metastases occurred in HepG2-control xenografts and HepG2-miR-26a (up-regulation miR-26a) xenografts based on quantification of bioluminescence. **d** Immunohistochemistry revealed that E-cadherin expression was decreased and the expression level of N-cadherin and vimentin was increased in HCCLM3-anti-miR-26a tumors compared to HCCLM3-controls. In HepG2-miR-26a tumors, EMT markers were inhibited compared to HepG2-controls. In addition, EZH2 expression was inversely correlated with expression of E-cadherin in HCCLM3 and HepG2 tumors. Representative images are shown at ×200. *P < 0.05 compared to control

To explore the role of miR-26a in tumor growth and metastasis, orthotopic nude mouse models were established with HepG2 and HCCLM3 cell lines. Mice with HCCLM3-anti-miR-26a tumors had lower body weights (18.6 ± 2.3 vs 21.2 ± 1.5 g 36 days after model establishment, *P* = 0.037) and larger tumors (973.5 ± 334.2 vs 515.6 ± 165.4 mm^3^, *P* = 0.019, Fig. 3a) than those with HCCLM3-control tumors. Mice with HepG2-miR-26a tumors had a higher body weight (19.9 ± 2.4 vs 16.7 ± 1.0 g 36 days after model establishment, *P* = 0.026) and smaller tumors (252.6 ± 98.1 vs 393.7 ± 107.5 mm^3^, *P* = 0.039, Fig. 3b) compared to those with HepG2-control tumors. Moreover, more pulmonary metastatic foci occurred in the HCCLM3-anti-miR-26a group than in the HCCLM3-control group (6.8 ± 1.7 vs 2.3 ± 1.0, *P* = 0.001, Fig 3c), which was shown in both bright field (b) and fluorescence (f) imaging. No pulmonary metastases were detected in the mouse models established with HepG2, a hepatoma cell line without potential for lung metastasis [26], including HepG2-miR-26a and HepG2-control cells (Fig. 3c). We further examined the expression of E-cadherin, N-cadherin, and vimentin in the orthotopic tumor samples. In the HCCLM3 groups, immunohistochemistry revealed typical membranous E-cadherin expression in the cell-cell contacts in the HCCLM3-control group, with low expression of N-cadherin and vimentin. In contrast, HCCLM3-anti-miR-26a tumors showed a significant reduction of E-cadherin expression, with up-regulated expression of N-cadherin and vimentin (the area of positive staining, 17,245.5 ± 5560.9 vs 41,578.8 ± 8220.3; 90,067.7 ± 12,891.1 vs 36,617.1 ± 5303.3; 27,053.1 ± 3910.9 vs 11,537.5 ± 2455.4; *P* < 0.05 for all, Fig. 3d). In the HepG2 groups, higher expression of E-cadherin and lower expression of N-cadherin and vimentin were found in the HepG2-miR-26a group in comparison to those in the HepG2-control group (the area of positive staining, 89,588.7 ± 10,305.1 vs 39,017.3 ± 6326.9; 18,865.1 ± 3244.4 vs 88,272.7 ± 9656.7; 16,910.5 ± 3601.1 vs 45,127.5 ± 3593.1; *P* < 0.05 for all, Fig. 3d). These results indicate that miR-26a significantly inhibited tumor growth and lung metastasis in vivo, along with down-regulating EMT-related makers. Furthermore, in HCCLM3-control tumors, the expression of EZH2 was lower than that in HCCLM3-anti-26a tumors (the integrated optical density [IOD] of positive staining, 13,744.9 ± 2210.5 vs 30,349.9 ± 4671.7, *P* < 0.005, Fig. 3d). In the HepG2 groups, the expression of EZH2 in HepG2-control group was significantly higher than that in the HepG2-miR-26a group (the IOD of positive staining, 26,594.3 ± 3067.9 vs 13,511.8 ± 2319.9, *P* < 0.005, Fig. 3d). These results indicate that miR-26a expression inhibited tumor progression in vivo and targeting EZH2-related EMT process may have been one of the main mechanisms.

### miR-26a expression was consistent with E-cadherin expression and inversely correlated with EZH2 expression in human HCC tissue

**Fig. 4:**
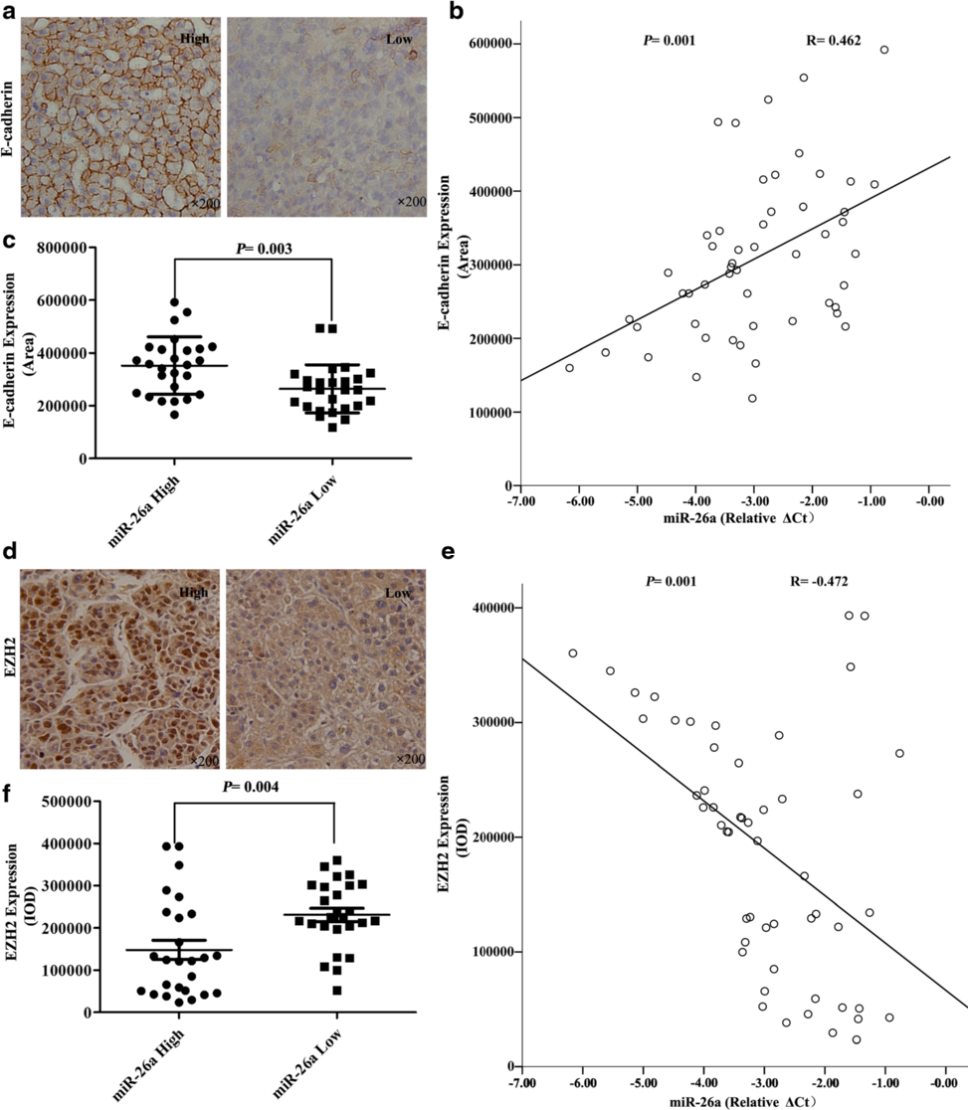
The relationships between miR-26a expression and the expression of EZH2 and E-cadherin in human HCC tissue. **a** A HCC tissue microarray was used to analyze E-cadherin expression using immunohistochemistry. Representative images are shown (×200). **b** miR-26a was positively associated with intratumoral E-cadherin expression (*R* = 0.462, *P* = 0.001). **c** Patients with high miR-26a expression had significantly higher E-cadherin expression compared with those with low miR-26a expression (*P* = 0.003). **d** Representative images of EZH2 staining are shown (×200). **e** An inverse association between miR-26a and EZH2 expression were found (*R* = −0.472, *P* = 0.001). **f** Patients with high miR-26a expression had significantly lower EZH2 expression compared with those with low miR-26a expression (*P* = 0.004)

To investigate the relationships between miR-26a expression and E-cadherin or EZH2 expression, we examined miR-26a expression in 52 HCC patients from 67 HCC patients with expression of E-cadherin and EZH2. miR-26a expression data were obtained in our previous study [17]. Representative images of immunostaining for E-cadherin are shown in Fig. 4a. E-cadherin expression was found to be positively associated with miR-26a expression (*R* = 0.462, *P* = 0.001, Fig. 4b). Using a median value of miR-26a expression as a cutoff point, we divided the 52 cases of HCC patients into two groups. Patients with high miR-26a expression had significantly higher E-cadherin expression compared with those with low miR-26a expression (the area of positive staining, 352,159.7 ± 108,722.0 vs 264,432.9 ± 91,518.7 *P* = 0.003, Fig 4c). In addition, EZH2 expression (Fig. 4d) was found to be inversely associated with miR-26a expression (*R* = −0.472, *P* = 0.001, Fig. 4e). Patients with high miR-26a expression had significantly lower EZH2 expression than those with low miR-26a expression (the area of positive staining, 148,246.9 ± 115,257.0 vs 231,286.3 ± 79,644.3, *P* = 0.004, Fig. 4f). The results further support an inverse relationship between miR-26a and EZH2 expression in human HCC tissue.

**Fig. 5:**
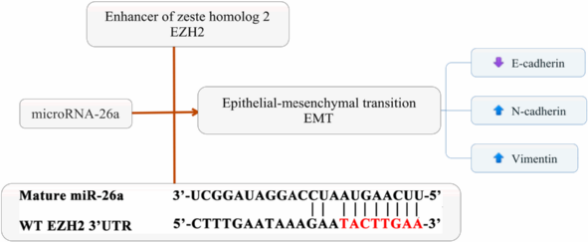
The proposed molecular mechanism was shown in a diagram

## Discussion

miR-26a plays a critical role in various types human cancers [17, 27, 28]. Although some studies have shown that it may function as an oncogene in malignant glioma, lung cancer, cholangiocarcinoma, and chronic lymphocytic leukemia [29–31], emerging evidence demonstrates that miR-26a may serve as a potential tumor suppressor in other malignant tumors [17, 32–35]. Previous study showed that miR-26a was down-regulated in HCC tumor samples with a poor prognosis, while high levels of miR-26a expression were associated with a good prognosis for patients who underwent curative liver resection [17]. Moreover, our previous study found that miR-26a inhibited HCC tumor growth and metastasis by modulating tumor microenvironment (e.g., inhibiting the tumor angiogenesis [15] and macrophage infiltration [14]). However, the underlying mechanisms associated with miR-26a in HCC invasion are still not fully understood. Here, we first demonstrated that miR-26a inhibits the EMT process by regulating EZH2 expression in HCC through a cell-autonomous mechanism (Fig. 5).

Metastasis and tumorigenesis compose a multi-step process [36], and a considerable amount of evidence indicates that EMT is responsible for converting noninvasive tumor cells into cells with metastatic potential, enabling invasion into the basement membrane and the vascular system, survival in the circulation, and extravasion at a distant secondary site [7, 37]. In addition, EMT has been demonstrated to significantly contribute to liver fibrosis, which provides a pre-metastatic niche or metastatic-supportive microenvironments [7, 38–40]. Various studies have demonstrated the critical role of miRNAs in EMT through regulation of E-cadherin expression [41, 42]. Moreover, some reports have found that miR-26a strongly reduces the expression of EZH2 in some tumors [23, 34, 43]. EZH2 is one catalytic subunit of the polycomb repressive complex 2, a transcriptional repressor of target genes. Overexpression of EZH2 was found to correlate with tumor aggressiveness, metastasis, and poor prognosis in numerous cancers [20, 21], including HCC [22, 44]. Furthermore, EZH2-mediated E-cadherin expression in some tumors [19, 45]. In the present study, miR-26a was found to be inversely correlated with EZH2 expression in the HCC cells and HCC tissues, and overexpression of miR-26a decreased the luciferase reporter activity of the wild-type 3′-UTR of EZH2 but not the mutant 3′-UTR. More importantly, the effects of miR-26a modulation on EMT in HCC were affected by the EZH2 siRNA. The data support that miR-26a could inhibit EMT by repressing the expression of EZH2. These results accord with findings in nasopharyngeal and oral squamous cell carcinomas [19, 23–25].

This study has some potential limitations. Our results showed that miR-26a inhibited EMT through targeting EZH2 expression. However, they did not exclude other signal pathways that may modulate EMT and could be mediated by miR-26a. Our preliminary study did not explore the effect of the HCC environment on miR-26a or EMT.

Since EMT has been critically discussed as the key process in tumor aggressiveness and metastasis [46], our findings in the present study demonstrate that miR-26a as a suppressive miRNA could inhibit tumor metastasis and invasion partly by impeding EMT through repression of EZH2. The data also suggest that miR-26a could be a marker and potential therapeutic target in HCC patients in the future.

## Conclusions

In conclusion, in view of the anti-invasive effects of miR-26a on tumor cells, therapeutic miR-26a delivery in the treatment of HCC deserves further investigation.

## Methods

### Cell line culture and morphological observation

The human HCC cell line HepG2 cells (obtained from the Cell Bank of the Chinese Academy of Sciences, Shanghai, China) and HCCLM3 cells [47] (human HCC cell lines with high metastatic potential, established at Liver Cancer Institute, Fudan University, Shanghai, China) were cultured in Dulbecco’s modified Eagle’s medium (DMEM; Invitrogen, Carlsbad, CA) containing 10 % fetal bovine serum (FBS) and 100 U ml^−1^ penicillin and 50 mg ml^−1^ streptomycin. All cells were incubated at 37 °C with a humidified atmosphere of 5 % CO_2_. The morphology of the HCC cells was observed using phase microscopy (Leica).

### miRNA and transfection

As described in our previous study [18], to modify miR-26a expression levels in HCC cell lines, we obtained recombinant lentivirus vectors from Genechem (Shanghai, China) that included genes such as pre-miR-26a, the negative control precursor miRNA; anti-miRNA-locked nucleic acids (LNAs) against miR-26a; and the negative control of anti-miRNA-LNAs. These vectors, with their packaging vectors, were transfected into 293 T cells using Lipofectamine 2000 (Invitrogen). HepG2 cells or HCCLM3 cells were then transfected with virus following the manufacturer’s instructions, and Western blot and quantitative RT-PCR (qRT-PCR) were used to validate the transfection results.

### Western blot

Cells were lysed using cell lysis buffer (150 mM NaCl, 50 mM Tris-HCl, pH 8.0, 0.1 % SDS, 1 % Triton X-100) containing protease and phosphatase inhibitors. Equivalent amounts of whole cell extracts were subjected to SDS-PAGE gel and transferred to nitrocellulose membranes. The membranes were blocked with 5 % nonfat milk for 2 h and then incubated with the respective primary antibody overnight at 4 °C, followed by the incubation of the appropriate HRP-conjugated secondary antibody for 2 h at room temperature. Blots were visualized with an ECL detection kit (Pierce, IL) and analyzed using Quantity One 1-D Analysis Software (Bio-Rad, San Francisco, CA).

### Cell migration assay

Quantitative cell migration assays were performed using a chamber (Corning, Tewksbury. MA) with 8.0-μm polycarbonate filter inserts in 24-well plates as described before [18]. About 1 × 10^5^ cells per well were added to the upper chamber, and the lower chamber was filled with cell medium. Cells were allowed to migrate for 24 h at 37 °C. Non-migrated cells were removed from the upper chamber using a cotton swab, and the migrated cells were fixed with methanol, stained with crystal violet, and photographed under an inverted microscope. Migration was assessed by counting the number of stained cells from five random fields at ×200 magnification.

### Luciferase activity assay

HEK293T cells were seeded in a 96-well plate at 50 to 60 % confluence. After 24 h, cells were transfected with 50 ng of miR-26a expression vector, miR-26a inhibitor, control vector, or negative control. Cells were co-transfected with 10 ng of the wild-type (WT) or mutant 3′UTR of the target gene. Transfections were performed using 0.45 μl of Fugene (Promega, Madison, WI). Forty-eight hours after transfection, cells were collected and Renilla luciferase activity was measured using a dual-luciferase reporter system (Promega) and detected using an Orion II microplate luminometer (Berthold, Bad Wildbad, Germany). Luciferase reporter assays were performed in quadruplicate and repeated in three independent experiments.

### Xenograft model of HCC in nude mice

Male BALB/c nude mice (5 weeks old, weighting 18–20 g) were purchased from the Shanghai Institute of Materia Medica (Chinese Academy of Science) and housed under specific pathogen-free conditions. The experimental protocol was approved by the Shanghai Medical Experimental Animal Care Commission. The various cells (6 × 10^6^ cells) in normal saline were implanted by subcutaneous injection (200 μl per mouse) to obtain the subcutaneous tumors. The mice were euthanized after 4 weeks to obtain the tumor tissues, which were used in the orthotopic model. The xenograft HCC model was established by orthotopic implantation of histologically intact tumor tissue into the nude mouse liver in the previous literature [48]. The body weights of mice were measured by electronic balance every week. Five weeks later, the mice were euthanized and their body weights were compared using independent-sample *t* test. After their volume was measured, the tumors (removed from liver) were placed in a 4 % paraformaldehyde solution. The tumor volume was calculated according to the formula: tumor volume = (largest diameter × perpendicular height^2^)/2.

### Patients, specimens, and follow-up

In this study, HCC specimens used in the immunohistochemical assay were obtained randomly from patients who underwent radical resection of pathologically confirmed tumors from 1999 to 2003 at the Liver Cancer Institute and Zhongshan Hospital (Fudan University, Shanghai, China); these specimens have been described previously [14, 17, 18]. No patients received any preoperative anticancer treatment. The present research was approved by the research ethics committee of Zhongshan Hospital. A total of 67 cases were used in this study to examine EZH2 expression, with 52 of the 67 HCC patients expressing miR-26a. The miR-26a expression data were obtained from our previous study. All patients included were followed up until 2011, with a median observation time of approximately 60 months. All patients provided written informed consent to participate in this study.

### Statistical analysis

Data analysis was performed using SPSS 19.0 for Windows (SPSS Inc. Chicago, IL). Unpaired Student’s *t* test or Pearson’s correlation test was used to compare quantitative variables. *P* < 0.05 was considered statistically significant.

## Abbreviations

EMT: epithelial-mesenchymal transition
EZH2: enhancer of zeste homolog 2
HCC: hepatocellular carcinoma
IL-6: interleukin-6
miR-26a: microRNA-26a

## References

[1] Forner A, Llovet JM, Bruix J (2012). Hepatocellular carcinoma. Lancet.

[2] Torre LA, Bray F, Siegel RL, Ferlay J, Lortet-Tieulent J, Jemal A (2015). Global cancer statistics, 2012. CA Cancer J Clin.

[3] Zhou XD, Tang ZY, Yang BH, Lin ZY, Ma ZC, Ye SL (2001). Experience of 1000 patients who underwent hepatectomy for small hepatocellular carcinoma. Cancer.

[4] Waly RS, Yangde Z, Yuxiang C (2012). Hepatocellular carcinoma: focus on different aspects of management. ISRN Oncol.

[5] Poon D, Anderson BO, Chen LT, Tanaka K, Lau WY, Van Cutsem E (2009). Management of hepatocellular carcinoma in Asia: consensus statement from the Asian Oncology Summit 2009. Lancet Oncol.

[6] Tung-Ping PR, Fan ST, Wong J (2000). Risk factors, prevention, and management of postoperative recurrence after resection of hepatocellular carcinoma. Ann Surg.

[7] van Zijl F, Zulehner G, Petz M, Schneller D, Kornauth C, Hau M (2009). Epithelial-mesenchymal transition in hepatocellular carcinoma. Future Oncol.

[8] Thiery JP, Sleeman JP (2006). Complex networks orchestrate epithelial-mesenchymal transitions. Nat Rev Mol Cell Biol.

[9] Grunert S, Jechlinger M, Beug H (2003). Diverse cellular and molecular mechanisms contribute to epithelial plasticity and metastasis. Nat Rev Mol Cell Biol.

[10] Perl AK, Wilgenbus P, Dahl U, Semb H, Christofori G (1998). A causal role for E-cadherin in the transition from adenoma to carcinoma. Nature.

[11] Wells A, Yates C, Shepard CR (2008). E-cadherin as an indicator of mesenchymal to epithelial reverting transitions during the metastatic seeding of disseminated carcinomas. Clin Exp Metastasis.

[12] Sanchez-Tillo E, Liu Y, de Barrios O, Siles L, Fanlo L, Cuatrecasas M (2012). EMT-activating transcription factors in cancer: beyond EMT and tumor invasiveness. Cell Mol Life Sci.

[13] Di LG, Garofalo M, Croce CM (2014). MicroRNAs in cancer. Annu Rev Pathol.

[14] Chai ZT, Zhu XD, Ao JY, Wang WQ, Gao DM, Kong J (2015). microRNA-26a suppresses recruitment of macrophages by down-regulating macrophage colony-stimulating factor expression through the PI3K/Akt pathway in hepatocellular carcinoma. J Hematol Oncol.

[15] Yang X, Zhang XF, Lu X, Jia HL, Liang L, Dong QZ (2014). MicroRNA-26a suppresses angiogenesis in human hepatocellular carcinoma by targeting hepatocyte growth factor-cMet pathway. Hepatology.

[16] Yang X, Liang L, Zhang XF, Jia HL, Qin Y, Zhu XC (2013). MicroRNA-26a suppresses tumor growth and metastasis of human hepatocellular carcinoma by targeting interleukin-6-Stat3 pathway. Hepatology.

[17] Ji J, Shi J, Budhu A, Yu Z, Forgues M, Roessler S (2009). MicroRNA expression, survival, and response to interferon in liver cancer. N Engl J Med.

[18] Chai ZT, Kong J, Zhu XD, Zhang YY, Lu L, Zhou JM (2013). MicroRNA-26a inhibits angiogenesis by down-regulating VEGFA through the PIK3C2alpha/Akt/HIF-1alpha pathway in hepatocellular carcinoma. PLoS One.

[19] Wang C, Liu X, Chen Z, Huang H, Jin Y, Kolokythas A (2013). Polycomb group protein EZH2-mediated E-cadherin repression promotes metastasis of oral tongue squamous cell carcinoma. Mol Carcinog.

[20] Takawa M, Masuda K, Kunizaki M, Daigo Y, Takagi K, Iwai Y (2011). Validation of the histone methyltransferase EZH2 as a therapeutic target for various types of human cancer and as a prognostic marker. Cancer Sci.

[21] Varambally S, Dhanasekaran SM, Zhou M, Barrette TR, Kumar-Sinha C, Sanda MG (2002). The polycomb group protein EZH2 is involved in progression of prostate cancer. Nature.

[22] Gao SB, Xu B, Ding LH, Zheng QL, Zhang L, Zheng QF (2014). The functional and mechanistic relatedness of EZH2 and menin in hepatocellular carcinoma. J Hepatol.

[23] Lu J, He ML, Wang L, Chen Y, Liu X, Dong Q (2011). MiR-26a inhibits cell growth and tumorigenesis of nasopharyngeal carcinoma through repression of EZH2. Cancer Res.

[24] Yu L, Lu J, Zhang B, Liu X, Wang L, Li SY (2013). miR-26a inhibits invasion and metastasis of nasopharyngeal cancer by targeting EZH2. Oncol Lett.

[25] Wu Y, Zhang L, Zhang L, Wang Y, Li H, Ren X (2015). Long non-coding RNA HOTAIR promotes tumor cell invasion and metastasis by recruiting EZH2 and repressing E-cadherin in oral squamous cell carcinoma. Int J Oncol.

[26] Zhang N, Wang L, Chai ZT, Zhu ZM, Zhu XD, Ma DN (2014). Incomplete radiofrequency ablation enhances invasiveness and metastasis of residual cancer of hepatocellular carcinoma cell HCCLM3 via activating beta-catenin signaling. PLoS One.

[27] Jansen MP, Reijm EA, Sieuwerts AM, Ruigrok-Ritstier K, Look MP, Rodriguez-Gonzalez FG (2012). High miR-26a and low CDC2 levels associate with decreased EZH2 expression and with favorable outcome on tamoxifen in metastatic breast cancer. Breast Cancer Res Treat.

[28] Zhang B, Liu XX, He JR, Zhou CX, Guo M, He M (2011). Pathologically decreased miR-26a antagonizes apoptosis and facilitates carcinogenesis by targeting MTDH and EZH2 in breast cancer. Carcinogenesis.

[29] Huse JT, Brennan C, Hambardzumyan D, Wee B, Pena J, Rouhanifard SH (2009). The PTEN-regulating microRNA miR-26a is amplified in high-grade glioma and facilitates gliomagenesis in vivo. Genes Dev.

[30] Zhang J, Han C, Wu T (2012). MicroRNA-26a promotes cholangiocarcinoma growth by activating beta-catenin. Gastroenterology.

[31] Zou ZJ, Fan L, Wang L, Xu J, Zhang R, Tian T (2015). miR-26a and miR-214 down-regulate expression of the PTEN gene in chronic lymphocytic leukemia, but not PTEN mutation or promoter methylation. Oncotarget.

[32] Calin GA, Croce CM (2006). MicroRNA signatures in human cancers. Nat Rev Cancer.

[33] Chang TC, Yu D, Lee YS, Wentzel EA, Arking DE, West KM (2008). Widespread microRNA repression by Myc contributes to tumorigenesis. Nat Genet.

[34] Ciarapica R, Russo G, Verginelli F, Raimondi L, Donfrancesco A, Rota R (2009). Deregulated expression of miR-26a and Ezh2 in rhabdomyosarcoma. Cell Cycle.

[35] Kota J, Chivukula RR, O’Donnell KA, Wentzel EA, Montgomery CL, Hwang HW (2009). Therapeutic microRNA delivery suppresses tumorigenesis in a murine liver cancer model. Cell.

[36] Steeg PS (2006). Tumor metastasis: mechanistic insights and clinical challenges. Nat Med.

[37] Creighton CJ, Chang JC, Rosen JM (2010). Epithelial-mesenchymal transition (EMT) in tumor-initiating cells and its clinical implications in breast cancer. J Mammary Gland Biol Neoplasia.

[38] Gunasinghe NP, Wells A, Thompson EW, Hugo HJ (2012). Mesenchymal-epithelial transition (MET) as a mechanism for metastatic colonisation in breast cancer. Cancer Metastasis Rev.

[39] Lee SJ, Kim KH, Park KK (2014). Mechanisms of fibrogenesis in liver cirrhosis: the molecular aspects of epithelial-mesenchymal transition. World J Hepatol.

[40] Lamouille S, Xu J, Derynck R (2014). Molecular mechanisms of epithelial-mesenchymal transition. Nat Rev Mol Cell Biol.

[41] Korpal M, Kang Y (2008). The emerging role of miR-200 family of microRNAs in epithelial-mesenchymal transition and cancer metastasis. RNA Biol.

[42] Gregory PA, Bert AG, Paterson EL, Barry SC, Tsykin A, Farshid G (2008). The miR-200 family and miR-205 regulate epithelial to mesenchymal transition by targeting ZEB1 and SIP1. Nat Cell Biol.

[43] Alajez NM, Shi W, Hui AB, Bruce J, Lenarduzzi M, Ito E (2010). Enhancer of Zeste homolog 2 (EZH2) is overexpressed in recurrent nasopharyngeal carcinoma and is regulated by miR-26a, miR-101, and miR-98. Cell Death Dis.

[44] Zhao H, Xu Y, Mao Y, Zhang Y (2013). Effects of EZH2 gene on the growth and migration of hepatocellular carcinoma HepG2 cells. Hepatobiliary Surg Nutr.

[45] Sun NX, Ye C, Zhao Q, Zhang Q, Xu C, Wang SB (2014). Long noncoding RNA-EBIC promotes tumor cell invasion by binding to EZH2 and repressing E-cadherin in cervical cancer. PLoS One.

[46] Thompson EW, Newgreen DF, Tarin D (2005). Carcinoma invasion and metastasis: a role for epithelial-mesenchymal transition. Cancer Res.

[47] Li Y, Tang ZY, Ye SL, Liu YK, Chen J, Xue Q (2001). Establishment of cell clones with different metastatic potential from the metastatic hepatocellular carcinoma cell line MHCC97. World J Gastroenterol.

[48] Sun FX, Tang ZY, Lui KD, Ye SL, Xue Q, Gao DM (1996). Establishment of a metastatic model of human hepatocellular carcinoma in nude mice via orthotopic implantation of histologically intact tissues. Int J Cancer.

